# New Design Method for Fabricating Multilayer Membranes Using CO_2_-Assisted Polymer Compression Process

**DOI:** 10.3390/molecules25245786

**Published:** 2020-12-08

**Authors:** Takafumi Aizawa

**Affiliations:** Research Institute for Chemical Process Technology, National Institute of Advanced Industrial Science and Technology, 4-2-1 Nigatake, Miyagino-ku, Sendai 983-8551, Japan; t.aizawa@aist.go.jp; Tel.: +81-22-237-5211

**Keywords:** CO_2_-assisted polymer compression, multilayer porous membrane, deep learning, carbon dioxide, process simulation

## Abstract

It was verified that deep learning can be used in creating multilayer membranes with multiple porosities using the CO_2_-assisted polymer compression (CAPC) method. To perform training while reducing the number of experimental data as much as possible, the experimental data of the compression behavior of two layers were expanded to three layers for training, but sufficient accuracy could not be obtained. However, the accuracy was dramatically improved by adding the experimental data of the three layers. The possibility of only simulating process results without the necessity for a model is a merit unique to deep learning. Overall, in this study, the results show that by devising learning data, deep learning is extremely effective in designing multilayer membranes using the CAPC method.

## 1. Introduction

Polymers, especially thermoplastics, are used for many purposes due to their ease of molding [1,2]. However, the fabrication of polymer materials is a process that requires a certain amount of energy for processing, as it involves heating and then cooling. I previously devised the CO_2_-assisted polymer compression (CAPC) method, in which polymers are plasticized with CO_2_ and then crimped so that they can be processed without applying heat [3]. Thus, this process is more energy-efficient than the heat-pressing process, and it contributes a key element of the Sustainable Development Goals (SDGs), “Target 9.4: Upgrade all industries and infrastructures for sustainability”, which aims at building sustainable societies [4].

CAPC is a method of plasticizing and crimping polymer fibers under the existence of CO_2_, and it can be realized at room temperature. Although CAPC is a high-pressure process, CO_2_ can be introduced by simply opening and closing a valve without using a pump. It is known that CO_2_ dissolves in polymers [5,6,7] and that polymers are plasticized by dissolving CO_2_ in their amorphous parts [8,9,10]. In the case that a plasticized polymer is a fibrous polymer, bonding is generated, as the fibers are overlapped and strongly pressed. The CAPC method produces porous materials by creating this point-bonding everywhere in the fibers, and the used CO_2_ is a low-toxicity substance that is also used in foods. Additionally, since CO_2_ does not remain in the created polymers when using the CAPC method, there is no concern about contamination, so this process can also be used in the medical and food industries. Among polymer fibers, nonwoven fabrics are mass-produced and inexpensive fabrics [11,12], and CAPC products made from this material can be applied not only to some high-value-added products but also to mass-produced products. In a previous report, the adhesive strength and penetration strength of a CAPC porous material was evaluated, and it was found that the adhesive strength was in good agreement with the experimental results, considering the aggregate of point bonding [13]. The control of porosity and pore size was examined, and it was found that increasing the compression rate reduced both the porosity and pore size [14]. In the CAPC procedure, since the compressibility can easily be changed by the press position, the characteristics of the porosity and pore size of a CAPC porous material can be controlled by the process conditions. The porosity and pore size cannot be controlled independently; if the porosity is determined, the pore size is also determined. When a polyethylene terephthalate nonwoven fabric with a fiber diameter of 8 μm was used, the pore size decreased from 8 to 1 μm as the porosity decreased from 0.32 to 0.07 [14]. It has also been found that it is easy to carry a drug between sheets and that the sustained release of the drug can easily be controlled by changing the number of sheets and the compression rate [15]. Regarding the productivity concerns due to batch production, producing multiple products with a single press was successful, and further study is being conducted on the necessary procedures for mass production [16].

Porous materials based on nonwoven fabric are used in various components, such as filters [17], with recent research investigating air filters [18] and solution filters [19]. There is also research on the applications of functional materials, such as battery separators [20]. For these porous materials, high functionality is a major theme. When considering filter applications, low pressure loss, a high particle capture rate, and a long life are key to high functionality. With regard to pressure loss, porosity exerts a strong influence, and CAPC porous materials are no exception [21]. A multilayer membrane with porosity and pore size gradients represents one possible solution to this problem.

Additionally, since CAPC products are porous materials with through holes, where the gaps between the fibers are pores, filters are considered to be among their applications. Therefore, the possibility of producing multilayer filters was examined, and it was found that it is possible to produce them by creating thick porous materials in the first step and then stacking and compressing them in the second step [22]. It was difficult to predict the thickness of each layer of the product, because each layer was not compressed in the same ratio during the compression process, and the softer layers were compressed more compared with harder layers. Moreover, in the design of these multilayer filters, it was found that the compression-after-expansion model is consistent with the experimental results. The initial expansion was modeled by determining an appropriate expansion value through an experiment, which was then incorporated into the model. Finally, the initial expansion value was adjusted as a parameter by fitting. The compression was modeled by incorporating the longitudinal modulus of the porous polymer material plasticized by CO_2_ as a function of density. Every time the raw material is changed, evaluating the initial expansion and the longitudinal modulus for building a new model is a laborious operation, so if a method for building a model can be found without using the initial expansion, the search for process conditions would be easier.

Artificial intelligence is, today, used in various fields of big data analysis, such as image analysis [23,24]. Even in chemistry fields, which to date, have primarily been experimental, studies on material informatics have begun [25,26,27]. Some artificial intelligence applications in chemistry fields are utilized in material design [28,29] and in analyzing various materials [30]. The background of the increase in these studies is the computing power improvements due to the development of GPGPU (general-purpose computing on graphics processing units) and the lowering of the hurdles of setting up computing environments due to the availability of free computing libraries. The advantage of deep learning is that it enables us to perform simulations without having to think about models. I was very interested in whether this advantage could be applied to the design of porous polymer materials using the CAPC method. However, deep learning often requires a huge amount of training data, which contradicts the preparation of huge amounts of training data to reduce the number of trial-and-error procedures necessary for creating target materials. Therefore, it is necessary to improve the accuracy of training data by adding data as needed and to construct initial training data with as few data as possible.

In this study, the objective was verifying whether it was possible to simulate a whole polymer fabrication process without using the initial expansion data and by learning the compression results through deep learning. In addition, it was studied whether it was possible to construct a deep learning model with a certain degree of accuracy by using as few training data as possible.

## 2. Results and Discussion

**Figure 1:**
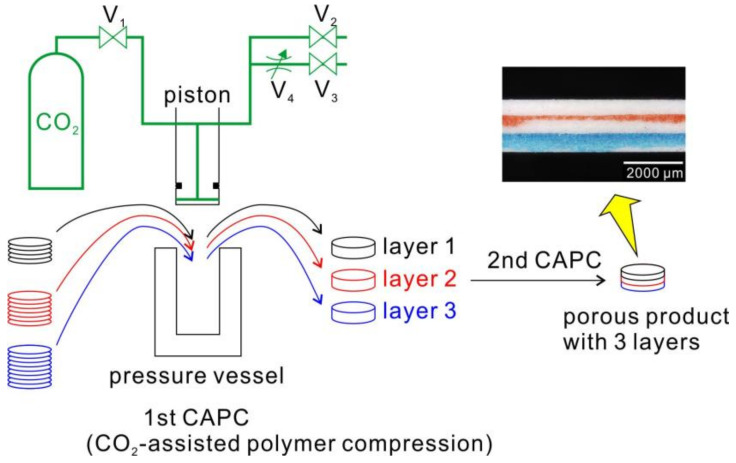
Fabrication process for the three-layer porous material (V_1_: introduction valve, V_2_: exhaust valve, V_3_: exhaust valve, V_4_: metering valve). The first CAPC process was carried out three times to produce layer 1, layer 2, and layer 3 samples; the second CAPC process was carried out once to construct the stacked sample. An optical microscope image of the cross-section of the porous product with three layers is shown in the figure.

In a previously published paper [22], the goal was to create a multilayered filter with three layers of equal thickness but different porosities via a two-step CAPC treatment (Figure 1). As an example of the product, an optical microscope image of the cross-section of the product created under particular conditions is shown in Figure 1. The surfaces of layer 2 and layer 3 were colored before the second CAPC to make the layer boundaries easier to see. To achieve this, I inferred the phenomena occurring in the high-pressure vessel and carefully explored the compression for two samples in order for humans to come up with a model based on this phenomenon. The results of the experiment, which were previously graphically presented, are presented as numerical values in Table 1.

Because this deep learning method requires three layers of data to be given as input values, creating three layers of data based on these two layers was considered. Deep learning was carried out using 10,000 generated training data. Since the number of training data was large for the total number of parameters (41,503), training was carried out without falling into over-training.

**Figure 2:**
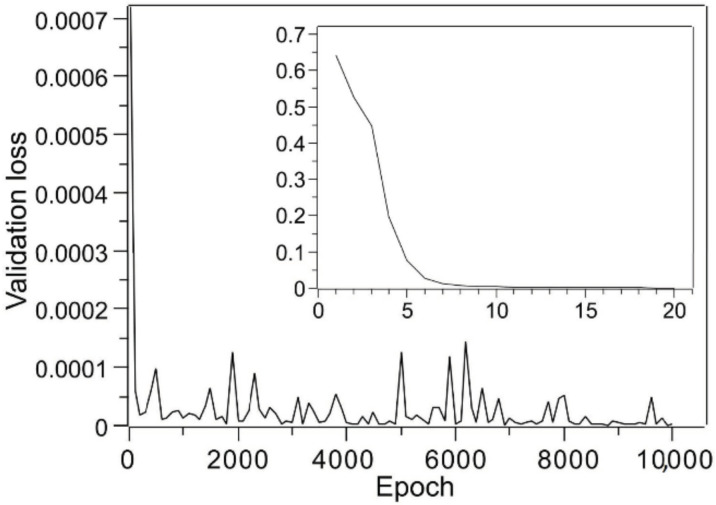
Changes in validation loss with respect to epochs based on training using the two-layer compression data shown in Table 1. The results for the first 20 epochs are displayed as an inset.

The validation of the training results was carried out using the compression results for the three measured layers in the previously reported study. Because the training results varied depending on the initialization state, the best result after ten training sessions was used to validate the results. The validation loss during a training session is shown in Figure 2. Table 2 shows the training results, which do not seem to properly correspond to the experimental results. The reason for this might be that the data were created by expanding the results of compressing two layers of the same weight into three layers, so training was carried out under the implicit rule of w_1i_ = w_2i_ + w_3i_ (or w_2i_ = w_1i_ + w_3i_, or w_3i_ = w_1i_ + w_2i_). It was assumed that learning was performed in the presence of the rules and that there was a problem with the reproducibility of the data that deviated from the rules.

**Figure 3:**
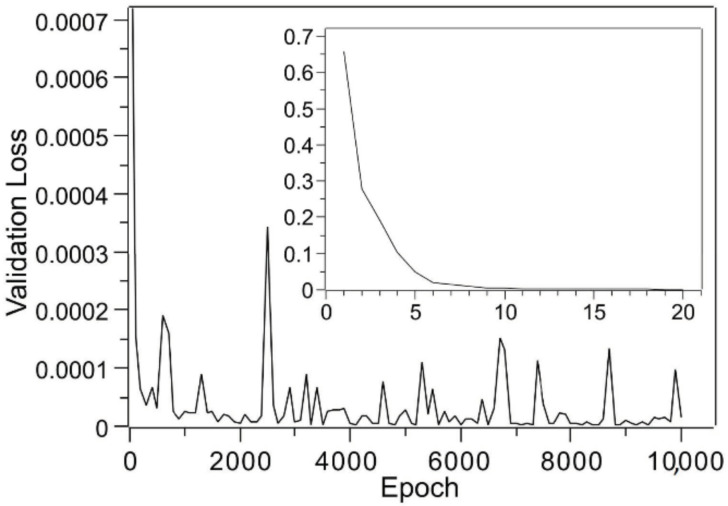
Changes in validation loss with respect to epochs based on training using both two-layer compression data (Table 1) and three-layer compression data (Table 3). The results for the first 20 epochs are displayed as an inset.

Therefore, the experimental results for the three samples that deviated from this rule were added, and it was determined experimentally whether learning was performed. In particular, experiments with 20 sheets (0.163 g total mass), 25 sheets (0.204 g total mass), and 30 sheets (0.244 g total mass) were conducted. The experimental results are provided in Table 3, which shows the average value for each of the four experiments. Table 4 shows the best results of 10 different training procedures, and it shows a dramatic improvement over the obtained training results for which only two-layer compression data were used. The validation loss during a training session is shown in Figure 3. The change in validation loss was similar to that in Figure 2. If the accuracy were improved to achieve this level, process design using deep learning could be applied to certain applications. Thus, the objective of this study, which was to establish a deep learning model with a certain degree of accuracy using as few training data as possible, was achieved. I believe that this method can be very effective in designing multilayer membranes using the CAPC method. Furthermore, the accuracy of the model can be improved by adding experimental training data.

## 3. Materials and Methods

### 3.1. Materials

The used sample was a nonwoven fabric (30 g m^−2^) with 8 μm-diameter fibers made of polyethylene terephthalate (model no.: TK3; Bell Polyester Products, Inc., Yamaguchi, Japan), and it was purchased from Nippon Nozzle Co., Ltd., Kobe, Japan. The nonwoven fabric was punched out to an 18 mm-diameter circle using a punch. The punched samples were transformed into samples for pressing by matching the numbers and weights. The prepared samples for this study were 20 sheets, 25 sheets, and 30 sheets with weights of 0.163, 0.204, and 0.244 g, respectively. In a previous study [22], the sample weights were both 0.521 g (64 sheets) for two-layer compression (Table 1), and the sample weights were 0.081 g (10 sheets), 0.098 g (12 sheets), and 0.122 g (15 sheets) for three-layer compression (Table 2 and Table 4).

### 3.2. Fabrication Procedure

The samples were prepared by the first-step CAPC for the porous polymer material, which constituted the layer. Then, the final layer structure was prepared by overlapping the porous materials with second-step CAPC treatment, and it was bonded while compressing the porous materials (Figure 1).

The outline of the CAPC apparatus is as follows. A piston and a pressure vessel were attached to a press machine (model no.: JP-1504; Janome Sewing Machine Co., Ltd., Hachioji, Japan). There was a CO_2_ channel in the center of the piston with a hole below the O-ring to seal the piston with the pressure vessel and to introduce CO_2_ into the pressure vessel or to exhaust CO_2_ from it. The import and export lines of the CO_2_ were connected to the top of the piston. The import line was connected through a ball valve (V_1_) from the CO_2_ cylinder, and the export line was divided into two lines: an instantaneous exhaust line for release to the atmosphere through a ball valve (V_2_) and a slow exhaust line with both a metering valve (V_4_) and a ball valve (V_3_).

In the first-step CAPC treatment, a predetermined amount of fiber sheets was set in the pressure vessel. Then, the piston was lowered to the CO_2_ introduction position, and the V_1_ valve was opened with the V_2_ and V_3_ valves closed to introduce the CO_2_. As the pressure vessel was initially filled with air, the air was diluted 60 times by the high CO_2_ pressure. Each time V_1_ was closed, V_2_ was opened, and the CO_2_ was exhausted along with the air. This process was repeated three times for the air in the container to be almost completely replaced by CO_2_. The CO_2_ was then introduced at vapor pressure by closing V_2_ and opening V_1_. After the CO_2_ was introduced, V_1_ was closed and the piston was moved to the press position and kept pressed for 10 s. Then, V_2_ was opened to exhaust the CO_2_. After the piston was raised, when the sample was removed from the pressure vessel, a porous polymer product was obtained with approximately the thickness of the press position. The CO_2_ introduction pressure was about 6 MPa, and the treatment was performed at room temperature.

Regarding the second-step CAPC process, Figure 1 shows the procedure of creating multilayer membranes. However, since it is difficult to measure the thickness of each layer when each layer is adhered to another one, separators were sandwiched between the layers, and CAPC treatment was performed under the condition that the layers did not adhere to each other. A sample with two separators sandwiched between three layers was set in a pressure vessel, the piston was lowered to the CO_2_ introduction position, the air was replaced with CO_2_ as in the first-step CAPC treatment, and then, CO_2_ was introduced. Then, the piston was lowered to the press position and pressed for 10 s. In a previous study, when processing multiple samples with separators, there was a tendency for the upper sample to become thicker when CO_2_ was instantly exhausted [16], so in the second CAPC treatment, V_3_ was opened for 30 s, and the V_4_ metering valve was used. After slow exhaustion through the valve, V_2_ was opened to release the CO_2_ to the atmosphere instantaneously. It is known that this treatment eliminates the phenomenon of the upper layer becoming thicker [16]. Regarding the thickness of each layer, the thickness of the central portion was measured using a micrometer caliper and a sample.

### 3.3. Deep Learning Model

A deep learning environment was constructed with Anaconda3 2019.7 [31] on a Windows 10 laptop PC with an NVIDIA GeForce RTX 2070. The Python version used in this study was 3.6.9 [32]. The model was built with Keras 2.2.4 [33] using TensorFlow 1.14.0 [34] as the backend, and the computation was performed in GPGPU via CUDA 10.0 [35] and cuDNN 10.0 [36].

Machine learning was carried out using a neural network model. First, the input and output layers were determined. The input layer was defined as the weight and thickness of the top sample at the time of preparation, the weight and thickness of the middle sample, the weight and thickness of the bottom sample, and the overall thickness after compression. The output layer was defined as the thickness of the top layer, the thickness of the middle layer, and the thickness of the bottom layer after compression. The hidden layer was five layers of 100 units for each layer. The activation function was the ramp function (parameter “ReLU” in Keras) for all the layers, as all the weights and thicknesses were positive numbers. The parameter “Glorot_Uniform” was used for initialization in all the layers. A loss evaluation was performed for the mean squared error, which was indicated using the “MSE” parameter, and training was performed 10,000 times, with 100,000 training data available, specifying that 90% of the input data should be used for training and 10% for validation. The total number of parameters in the model was 41,503, all of which were used as training parameters. The parameter “Adam” was specified as an optimizer.

### 3.4. Procedure for Making Training Data from Two-Layer Compression Data

A case was considered in which the thicknesses of the two layers were x_1i_ and x_2i_ and the thicknesses after compression were x_1f_ and x_2f_, respectively. The total thickness x_f_ after compression was x_1f_ + x_2f_, and x_1i_ and x_2i_ were randomly selected from Table 1. Note that there are two combinations of x_1i_ and x_2i_ for the same row of data. In one combination, x_1i_ is larger, and in the other one, x_2i_ is larger. Thus, from Table 1, there are 46 possible combinations of x_1i_ and x_2i_. In order to create three-layer data from two-layer data, one layer must be split. Therefore, the splitting ratio, defined as the amount that determines the division ratio of one layer, was introduced. Considering the splitting ratio r (0 ≤ r ≤ 0.5), x_2i_ was divided into two layers: x_2i1_ = x_2i_ × r and x_2i2_ = x_2i_ × (1 − r). The compression results were also split by the ratio r: x_2f1_ = x_2f_ × r and x_2f2_ = x_2f_ × (1 − r). The x_1i_, x_2i1_, and x_2i2_ were randomly shuffled to create y_1i_, y_2i_, and y_3i_, respectively, and there were six shuffling combinations. The corresponding compression results were stored in y_1f_, y_2f_, and y_3f_. If the same combination of materials was used and the preparation thickness was halved, it was expected that the result would be halved as well. Therefore, the coefficients f (0 < f ≤ 1) were introduced to increase the variation of this preparation thickness, and z_1i_ = y_1i_ × f, z_2i_ = y_2i_ × f, z_3i_ = y_3i_ × f, z_1f_ = y_1f_ × f, z_2f_ = y_2f_ × f, and z_3f_ = y_3f_ × f were prepared. The introduction of these two factors of r and f made it possible to increase the number of training data beyond the fitting parameters. In practice, the weights w_1i_, w_2i_, and w_3i_ were also determined after considering the effects of r and f, and w_1i_, z_1i_, w_2i_, z_2i_, w_3i_, z_3i_, and t were included as input layers, where t is the total thickness after compression (t = z_1f_ + z_2f_ + z_3f_). The corresponding output layers were z_1f_, z_2f_, and z_3f_. I used this method to create 100,000 training data for deep learning based on Table 1. Note that 100,000 training data were prepared that did not have exactly the same data by recalculating the results determined by random numbers when they were the same.

### 3.5. Procedure for Making Training Data from Both Two-Layer and Three-Layer Compression Data

In the case of three-layer data, it was not necessary to split one layer to make three-layer data as in the case of the two-layer data. The initial thicknesses (x_1i_, x_2i1_, and x_2i2_) and thicknesses after compression (x_1f_, x_2f1_, and x_2f2_) were obtained from experimental data. Thus, only the process of making y_1i_, y_2i_, and y_3i_ through shuffling and the process of introducing the coefficients f to make z_1i_, z_2i_, and z_3i_ were performed. In other words, for the production of the data of Table 1 and Table 3 together, each datum was randomly chosen, and when the data from Table 1 were chosen, the data were produced using the same training-data-generation process described in Section 3.4. Moreover, when the data of Table 3 were chosen, the data were produced by shuffling the samples and taking into account the coefficients f. The 100,000 different training data, while making sure not to make the data the same, were created.

## 4. Conclusions

In this study, by using deep learning, I attempted to predict the thickness of each layer of three layers that were to be produced using the CAPC process without building a complex model and by simply learning the actual compression results. Though it was not sufficient to create three pseudo-compression results from two compression results, the addition of three compression results was found to result in a good simulation. In this paper, it was found that the experimental results could be explained by adding six three-layer experimental data points to the three-layer data extended from two layers, but additional data may be required depending on the target multilayer-membrane configuration. However, this paper suggests that it is possible to reduce the quantity of data required for initial training by using data extended from two layers as the foundational data. The possibility of only simulating process results without the necessity for a model is a merit unique to deep learning. This time, it was applied to compression in the presence of CO_2_, but it is believed that it can also be applied when forming multiple layers by compression, such as in heat pressing. Generally, when the number of training data can be increased by processing experimental data, deep learning is considered an effective method for selecting the process conditions, as it allows the prediction of process outcomes with fewer data by appropriately selecting training data. However, when increasing data, it is often the case that the data are increased based on a certain rule, so it is important to note that the learning result may not be an expected one because of that rule.

## Figures and Tables

**Table 1:** Thickness of each layer for the two-layer compression (Reference [22]).

Before Compression		After Compression	
Layer 1	Layer 2	Layer 1	Layer 2
Thickness (mm)	Thickness (mm)	Thickness (mm)	Thickness (mm)
5.001	4.001	4.140	3.866
5.000	4.000	3.589	3.408
5.000	4.001	3.038	2.963
5.000	4.001	2.547	2.459
5.001	4.000	2.298	2.228
5.000	4.001	2.028	1.980
5.000	3.003	3.853	3.123
5.000	3.002	3.145	2.843
5.000	3.002	2.565	2.443
5.000	3.002	2.287	2.236
4.999	3.003	2.033	1.979
5.000	2.000	3.749	2.243
5.001	2.000	2.849	2.164
5.000	1.999	2.458	2.061
5.001	2.000	2.125	1.871
4.000	3.002	3.119	2.859
4.000	3.002	2.532	2.471
4.000	3.002	2.276	2.239
4.000	3.002	2.016	1.992
4.000	1.998	2.864	2.153
4.000	1.999	2.461	2.055
3.999	1.999	2.118	1.898
3.003	2.000	2.112	1.906

The sample weight of all the layers is 0.521 g.

**Table 2:** Experimentally obtained thickness [22] for three-layer compression and deep learning prediction based on training using the two-layer compression data shown in Table 1.

Before Compression			After Compression					
			Experimental Results			Prediction by Deep Learning		
Layer 1	Layer 2	Layer 3	Layer 1	Layer 2	Layer 3	Layer 1	Layer 2	Layer 3
Thickness (mm)	Thickness (mm)	Thickness (mm)	Thickness (mm)	Thickness (mm)	Thickness (mm)	Thickness (mm)	Thickness (mm)	Thickness (mm)
0.696	0.695	0.694	0.501	0.589	0.711	0.577	0.612	0.626
0.697	0.647	0.600	0.535	0.602	0.658	0.609	0.614	0.579
0.740	0.573	0.535	0.608	0.604	0.586	0.673	0.575	0.551

The sample weights were 0.081, 0.098, and 0.122 g for layer 1, layer 2, and layer 3, respectively.

**Table 3:** Thickness of each layer for the three-layer compression (this work).

Before Compression			After Compression		
Layer 1	Layer 2	Layer 3	Layer 1	Layer 2	Layer 3
Thickness (mm)	Thickness (mm)	Thickness (mm)	Thickness (mm)	Thickness (mm)	Thickness (mm)
1.400	1.400	1.400	1.137	1.322	1.441
1.400	1.400	1.400	1.021	1.218	1.361
1.400	1.400	1.400	0.917	1.113	1.270
1.400	1.400	1.400	0.822	1.009	1.169
1.200	1.200	1.200	0.954	1.119	1.227
1.200	1.200	1.200	0.842	1.014	1.144

The sample weights were 0.163, 0.204, and 0.244 g for layer 1, layer 2, and layer 3, respectively.

**Table 4:** Experimentally obtained thickness [22] for three-layer compression and deep learning prediction based on training using both two-layer compression data (Table 1) and three-layer compression data (Table 3).

Before Compression			After Compression					
			Experimental Results			Prediction by Deep Learning		
Layer 1	Layer 2	Layer 3	Layer 1	Layer 2	Layer 3	Layer 1	Layer 2	Layer 3
Thickness (mm)	Thickness (mm)	Thickness (mm)	Thickness (mm)	Thickness (mm)	Thickness (mm)	Thickness (mm)	Thickness (mm)	Thickness (mm)
0.696	0.695	0.694	0.501	0.589	0.711	0.513	0.603	0.682
0.697	0.647	0.600	0.535	0.602	0.658	0.546	0.606	0.639
0.740	0.573	0.535	0.608	0.604	0.586	0.609	0.574	0.609

The sample weights were 0.081, 0.098, and 0.122 g for layer 1, layer 2, and layer 3, respectively.
